# Supplementary material for: Providing Diabetes Education through Phone Calls Assisted in the Better Control of Hyperglycemia and Improved the Knowledge of Patients on Diabetes Management
Source: Healthcare (Basel). 2023 Feb 10;11(4):528. doi: 10.3390/healthcare11040528 (PMC9957542; doi:10.3390/healthcare11040528)
Supplement: Supplementary file 1 [file healthcare-11-00528-s001.zip › Supplemental Figure 1a.pdf]

Supplemental Figure S1a,b

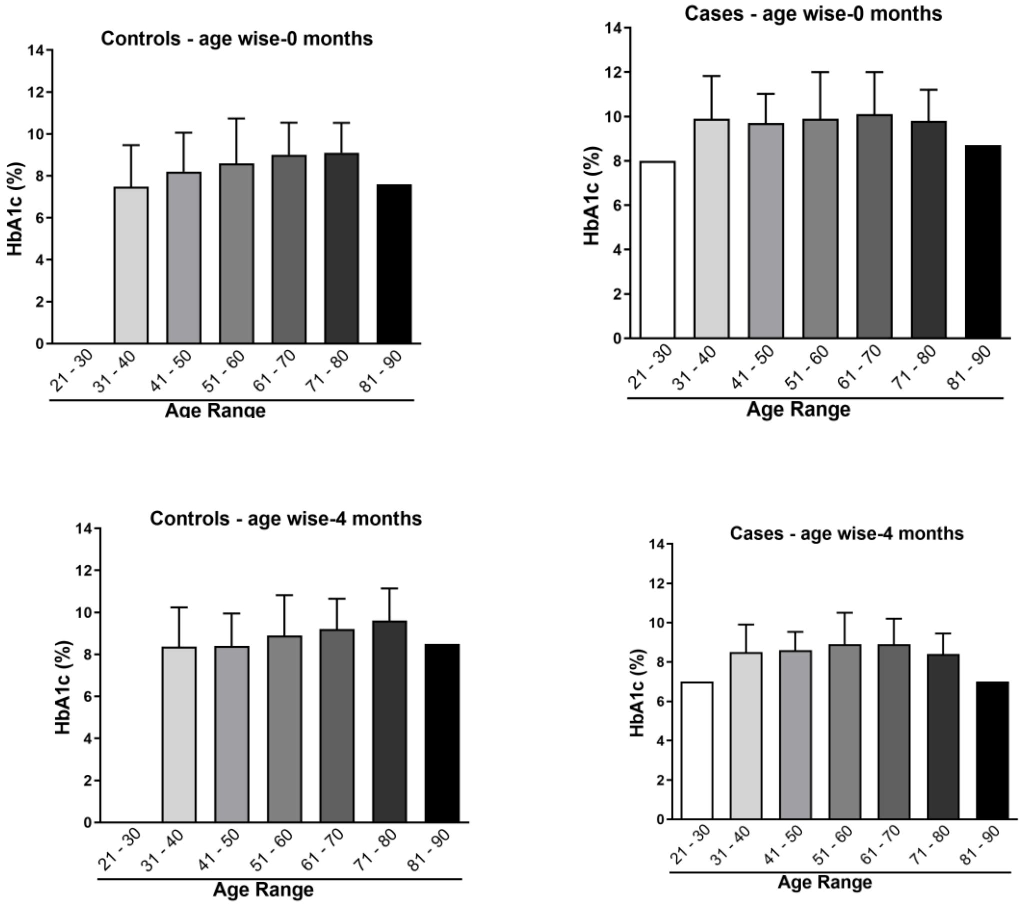

Supplemental Figure S1a: Age-wise distribution of study participants and their HbA1C (%) values in RCT: Analysis of age wise distribution of study participants showed that majority of them belong to the age group between 41-70 years in both control and cases. The HbA1C value in each age group category between the control and cases was not significant (P > 0.05 by Two-Way ANOVA Tukey's multiple comparison test).
