# Supplementary material for: Providing Diabetes Education through Phone Calls Assisted in the Better Control of Hyperglycemia and Improved the Knowledge of Patients on Diabetes Management
Source: Healthcare (Basel). 2023 Feb 10;11(4):528. doi: 10.3390/healthcare11040528 (PMC9957542; doi:10.3390/healthcare11040528)
Supplement: Supplementary file 1 [file healthcare-11-00528-s001.zip › Supplemental Figure 1b.pdf]

Supplemental Figure S1b

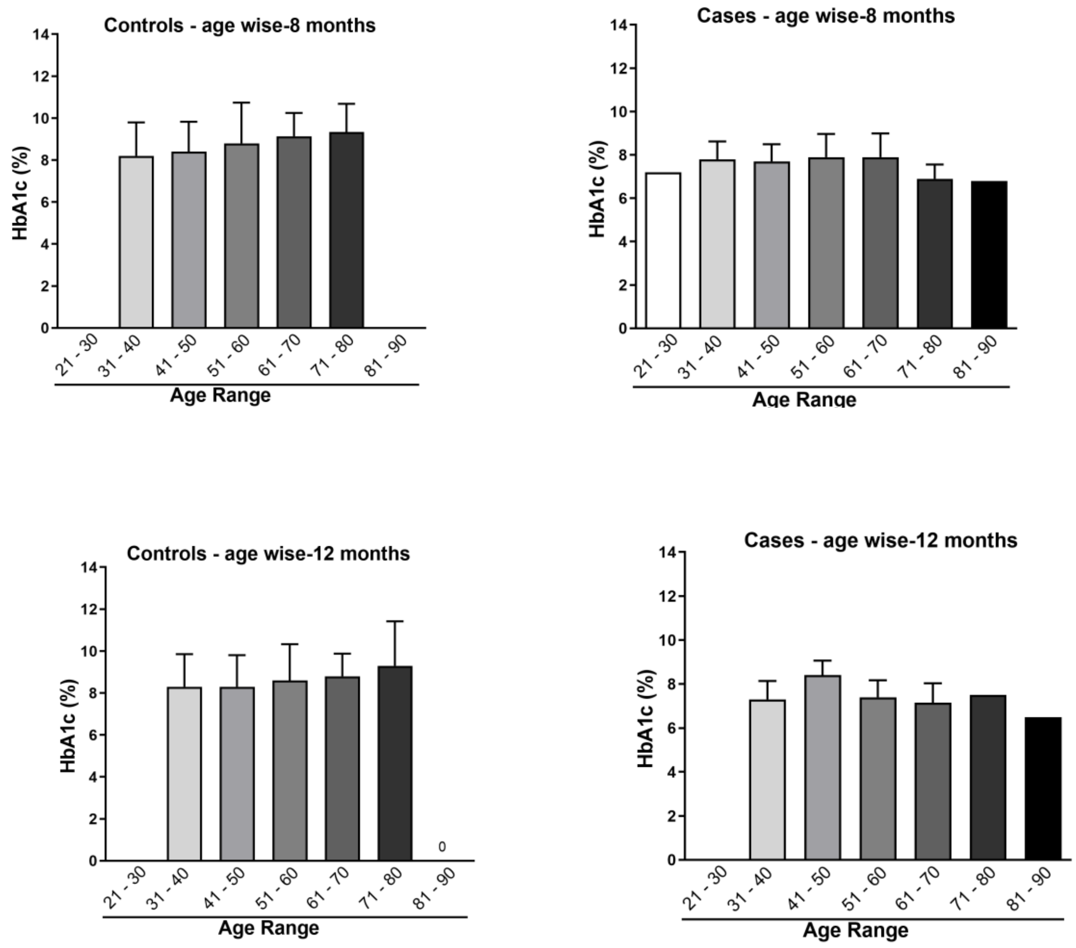

Supplemental Figure S1b: Variations in the number of study participants and their HbA1C (%) across different age groups in the RCT. Numbers above the bar shows the average HbA1C with standard deviation. Although there is no much variation in the number of study participants at each age group, a visible improvement in the HbA1C was observed in the case group compared to controls. The differences in HbA1C were significant in case group when compared to base line value, especially in the age groups
