# Supplementary material for: Providing Diabetes Education through Phone Calls Assisted in the Better Control of Hyperglycemia and Improved the Knowledge of Patients on Diabetes Management
Source: Healthcare (Basel). 2023 Feb 10;11(4):528. doi: 10.3390/healthcare11040528 (PMC9957542; doi:10.3390/healthcare11040528)
Supplement: Supplementary file 1 [file healthcare-11-00528-s001.zip › Supplemental information 10 - Knowledge score cards.pdf]

## Diabetes knowledge scores of study participants

| Sno | Date | Patient name | Scores for each question | | | | | | | | | | | Remarks |
|---|---|---|---|---|---|---|---|---|---|---|---|---|---|---|
| | | | Q1 | Q2 | Q3 | Q4 | Q5 | Q6 (Score) Answer | | Q7 | Q8 | Q9 | Q10 | |
| | | | | | | | | | | | | | | |
| | | | | | | | | | | | | | | |
| | | | | | | | | | | | | | | |
| | | | | | | | | | | | | | | |
| | | | | | | | | | | | | | | |
