# Supplementary material for: Providing Diabetes Education through Phone Calls Assisted in the Better Control of Hyperglycemia and Improved the Knowledge of Patients on Diabetes Management
Source: Healthcare (Basel). 2023 Feb 10;11(4):528. doi: 10.3390/healthcare11040528 (PMC9957542; doi:10.3390/healthcare11040528)
Supplement: Supplementary file 1 [file healthcare-11-00528-s001.zip › Supplemental information 2.2-Informed Consent Kannada.pdf]

ಈ ತಿಳಿಸಿದ ಒಪ್ಪಿಗೆಯು ಜೆಎಸ್‌ಎಸ್ ಆಸ್ಪತ್ರೆಗೆ ಬರುವ ರೋಗಿಗಳಿಗಾಗಿದ್ದು ಮತ್ತು Effectiveness of Self Management in Type2 Diabetic Patients through Ihformation Communication Technology (ICT) Interventions" ಎನ್ನುವ ಶೀರ್ಷಿಕೆಯೊಂದಿಗೆ ಸದರಿ ಸಂಶೋಧನೆಗಾಗಿ ಆಹ್ವಾನಿಸಿರುವವರಿಗಾಗಿದೆ.

ಪ್ರಧಾನ ತನಿಖೆದಾರರು : ಕನಕವಲ್ಲಿ ಕುಂಡೂರಿ
ಸಹಾಯಕ ಪ್ರಾಧ್ಯಾಪಕರು
ಆರೋಗ್ಯ ಪದ್ಧತಿ ನಿರ್ವಹಣಾ ಅಧ್ಯಯನ ವಿಭಾಗ
ಜಗದ್ಗುರು ಶ್ರೀ ಶಿವರಾತ್ರೀಶ್ವರ ವಿಶ್ವವಿದ್ಯಾನಿಲಯ,
ಮೈಸೂರು. 570015

ಮಾಹಿತಿ ಪುಟ

ಪರಿಚಯಃ

ನಾನು ಕನಕವಲ್ಲಿ ಕೆ. ಕುಂಡೂರಿ ಎಂಬ ಹೆಸರಿನವಳಾಗಿದ್ದು, ಜೆಎಸ್‌ಎಸ್ ವಿಶ್ವವಿದ್ಯಾನಿಲಯದಲ್ಲಿ ಕೆಲಸ ನಿರ್ವಹಿಸುತ್ತಿರುತ್ತೇನೆ. ನಾವು Effectiveness of Self Management in Type2 Diabetic Patients through Ihformation Communication Technology (ICT) Interventions ಎನ್ನುವ ಬಗ್ಗೆ ಸಂಶೋಧನೆಯನ್ನು ನಡೆಸುತ್ತಿದ್ದೇವೆ. ಸದರಿ ಸಂಶೋಧನೆಯಲ್ಲಿ ಪಾಲ್ಗೊಳ್ಳುವುದಕ್ಕಾಗಿ ನಾನು ನಿಮಗೆ ಆಹ್ವಾನ ನೀಡುವ ಮಾಹಿತಿಯನ್ನು ನೀಡುತ್ತಿದ್ದೇನೆ. ಇದರಲ್ಲಿ ಕೆಲವು ಪದಗಳು ನಿಮಗೆ ಅರ್ಥವಾಗದೇ ಇರಬಹುದು. ಅದನ್ನು ನೀವು ನನ್ನನ್ನು ಕೇಳಿದಲ್ಲಿ ನಾನು ನಿಮಗೆ ಅದನ್ನು ವಿವರಿಸುತ್ತೇನೆ. ಈ ಸಂಶೋಧನೆಯಲ್ಲಿ ಪಾಲ್ಗೊಳ್ಳುವ ಮುನ್ನ ನೀವು ಇಚ್ಛಿಸಿದಲ್ಲಿ, ನೀವು ನಿಮ್ಮ ಕುಟುಂಬದವರೊಂದಿಗೆ ಅಥವಾ ನಿಮ್ಮ ಸ್ನೇಹಿತರೊಂದಿಗೆ ಸಂಶೋಧನೆಯ ಕುರಿತಾಗಿ ಮಾತನಾಡಬಹುದಾಗಿದೆ.

ಸಂಶೋಧನೆಯ ಉದ್ದೇಶಃ

ಮಧುಮೇಹ ಎನ್ನುವುದು ಸಾಮಾನ್ಯ ಕಾಯಿಲೆಗಳಲ್ಲೊಂದಾಗಿದ್ದು, 2015 ರಲ್ಲಿನ ವಿಶ್ವ ಆರೋಗ್ಯ ವರದಿಯ ಪ್ರಕಾರ, ಭಾರತವು ವಿಶ್ವದ ಮಧುಮೇಹಿಗಳ ರಾಜಧಾನಿಯಾಗಿರುತ್ತದೆ. ಈ ಮಧುಮೇಹ ರೋಗದ ದುಷ್ಪರಿಣಾಮಗಳು ವಿಪರೀತ ಬಾಯಾರಿಕೆ, ತೂಕದಲ್ಲಿ ಇಳಿಕೆ, ಪದೇ ಪದೇ ಮೂತ್ರ ವಿಸರ್ಜನೆ, ಗಮನವಿರಿಸುವಲ್ಲಿ ತೊಂದರೆ, ಆಹಾರ ತೊಂದರೆಗಳು, ಹೃದಯ ಕಾಯಿಲೆಗಳು ಮತ್ತು ಪಾರ್ಶ್ವವಾಯು, ಕ್ಯಾಟರಾಕ್ಟ್ ಮತ್ತು ದೃಷ್ಟಿ ದೋಷಗಳಾಗಿರುತ್ತವೆ. ಈ ಕಾಯಿಲೆಯ ಹೊರೆಯು ಬರಿ ಜನಸಂಖ್ಯೆಯ ಉತ್ಪತ್ತಿಯ ಮೇಲೆ ಪರಿಣಾಮ ಬೀರುವುದಲ್ಲದೆ ದೇಶಗಳ ಮೇಲೆ ಹೆಚ್ಚುವರಿ ಆರೋಗ್ಯ ರಕ್ಷಣೆಗಾಗಿ ವೆಚ್ಚವನ್ನು ಭರಿಸಬೇಕಾಗುತ್ತದೆ. ಮಧುಮೇಹವನ್ನು ರೋಗಿಗಳಿಗೆ ಸೂಕ್ತ ತಿಳುವಳಿಕೆ ನೀಡುವುದರ ಮೂಲಕ ದೊಡ್ಡ ಮಟ್ಟಿಗೆ ನಿರ್ವಹಿಸಬಹುದಾಗಿದ್ದು ಮತ್ತು ಮದುಮೇಹದ ರೋಗಿಗಳಲ್ಲಿ ಕ್ರಮಬದ್ಧವಾಗಿ ಬದಲಾವಣೆಗಳನ್ನು ಉಸ್ತುವಾರಿಯೊಡನೆ ಅವರ ಆರೋಗ್ಯ ಸ್ಥಿತಿ ಮತ್ತು ಸ್ವಯಂರಕ್ಷಣೆಯನ್ನು ಬೆಂಬಲಿಸುವುದಾಗಿದೆ. ಮಧುಮೇಹವನ್ನು ನಿಯಂತ್ರಿಸುವುದಕ್ಕಾಗಿ ವಿಶ್ವದಲ್ಲೆಡೆ ಹಲವಾರು ಮಧುಮೇಹ ತಿಳುವಳಿಕೆ ಕಾರ್ಯಕ್ರಮಗಳನ್ನು ಆಯೋಜಿಸಲಾಗಿದೆ. ಹೆಚ್ಚುತ್ತಿರುವ ಮಾಹಿತಿ ಸಂವಹನ ತಂತ್ರಜ್ಞಾನವು ಆರೋಗ್ಯ ರಕ್ಷಣೆಯ ಮಾಹಿತಿಯನ್ನು ಕೈ ಬೆರಳ ತುದಿಯಲ್ಲಿಯೇ ದೊರಕುವಂತೆ ಮಾಡಿದೆ. ಪ್ರಸ್ತುತ ಸಂಶೋಧನೆಯಲ್ಲಿ ನಾವು ರೋಗಿಗಳಿಗೆ ಒದಗಿಸುವ ಮಾಹಿತಿ ಸಂವಹನ ತಂತ್ರಜ್ಞಾನದ ಸಲಕರಣೆಗಳ ಮೂಲಕ ಮಧುಮೇಹವನ್ನು ನಿರಂತರವಾಗಿ ಸ್ವಯಂ ನಿರ್ವಹಿಸಬಹುದಾಗಿದೆ.

ಸಂಶೋಧನೆಯ ವಿವಿಧ ಹಸ್ತಕ್ಷೇಪಗಳುಃ

ಭಾಗವಹಿಸುವವರು ಈ ಅಧ್ಯಯನಕ್ಕಾಗಿ ಒಪ್ಪಿಗೆ ನೀಡಿದ ನಂತರ ಅವರು ನೀಡಿರುವ ಮೊಬೈಲ್ ಸಂಖ್ಯೆಗೆ ಮದುಮೇಹ ಶಿಕ್ಷಣ ಕುರಿತಾದ ಸಂದೇಶಗಳು ಮತ್ತು ಕರೆಗಳನ್ನು ಪಡೆಯುವವರಾಗಿದ್ದು ಅವರನ್ನು ಗ್ಲೈಸಮಿಕ್ ನಿಯತಾಂಕಗಳನ್ನು ಮಾನಿಟರ್ ಮಾಡಲು ಪ್ರೇರೇಪಿಸಲಾಗುವುದು. ಭಾಗವಹಿಸುವವರ ಕಾಲಿಕ ಗ್ಲೈಸಮಿಕ್ ರೀಡಿಂಗ್‌ಗಳನ್ನು ಸಂಗ್ರಹಿಸಿ ಮಾಹಿತಿ ಸಂವಹನ ತಂತ್ರಜ್ಞಾನ ಸಲಕರಣೆಗಳ ಮೂಲಕ ಸ್ವಯಂ ನಿರ್ವಹಿಸುವ ಪರಿಣಾಮಗಳ ಬಗ್ಗೆ ನಿರ್ಣಯಿಸಲಾಗುವುದು.

ಭಾಗವಹಿಸುವಿಕೆ ಆಯ್ಕೆ

ಜೆಎಸ್‌ಎಸ್ ಆಸ್ಪತ್ರೆಗೆ ಬರುವ ಮಧುಮೇಹ ರೋಗಿಗಳನ್ನು ಸದರಿ Effectiveness of Self Management in Type2 Diabetic Patients through Ihformation Communication Technology (ICT) Intervention ಸಂಶೋಧನೆಯಲ್ಲಿ ಭಾಗವಹಿಸಲು ಆಹ್ವಾನಿಸಲಾಗುವುದು.

ಸ್ವಯಂಪ್ರೇರಿತ ಭಾಗವಹಿಸುವಿಕೆಃ

ಈ ಸಂಶೋಧನೆಯಲ್ಲಿ ನಿಮ್ಮ ಭಾಗವಹಿಸುವಿಕೆ ಸ್ವಯಂಪ್ರೇರಿತವಾಗಿರುತ್ತದೆ. ಭಾಗವಹಿಸಬೇಕೋ ಬೇಡವೋ ಎನ್ನುವುದು ನಿಮಗೆ ಬಿಟ್ಟ ಆಯ್ಕೆ. ನೀವು ಭಾಗವಹಿಸುವಿರೋ ಇಲ್ಲವೋ, ಆದರೆ ನಿಮಗೆ ಈ ಔಷಧಾಲಯದಲ್ಲಿ ನೀಡಲಾಗುವ ಎಲ್ಲ ಸೇವೆಗಳು ಎಂದಿನಂತೆ ಮುಂದುವರಿಯುವುವು ಹಾಗೂ ಇದರಲ್ಲಿ ಯಾವುದೇ ಬದಲಾವಣೆಗಳಿರುವುದಿಲ್ಲ. ಒಮ್ಮೆ ಒಪ್ಪಿ ನಂತರ ನೀವು ಭಾಗವಹಿಸುವಿಕೆಯಿಂದ ಹಿಂದೆಗೆಯಲು ಮನಸ್ಸು ಮಾಡಿದಲ್ಲಿ, ಹಾಗೆ ಮಾಡಬಹುದಾಗಿದೆ.

ಅವಧಿಃ

ಒಟ್ಟಾರೆ ಈ ಸಂಶೋದನೆಯ ಅವದಿ 12 ತಿಂಗಳುಗಳಾಗಬಹುದು. ಕಾಲ ಕಾಲಕ್ಕೆ ಗ್ಲೈಸಮಿಕ್ ನಿಯತಾಂಕಗಳನ್ನು ಭಾಗವಹಿಸುವವರಿಂದ ಅಧ್ಯಯನಕ್ಕಾಗಿ ಸಂಗ್ರಹಿಸಲಾಗುವುದು.

ಗೋಪ್ಯತೆ:

ಈ ಸಂಶೋಧನೆಯಿಂದ ನಾವು ಸಂಗ್ರಹಿಸಲ್ಪಡುವ ಮಾಹಿತಿಯನ್ನು ಗೋಪ್ಯವಾಗಿ ಇರಿಸಲಾಗುವುದು. ಸಂಶೋಧನೆಯ ಸಮಯದಲ್ಲಿ ಸಂಗ್ರಹಿಸಿದ ಮಾಹಿತಿಯನ್ನು ಸಂಶೋಧಕರು ಅವಲೋಕಿಸಬಹುದಾಗಿರುತ್ತದೆ.

ಯಾರನ್ನು ಸಂಪರ್ಕಿಸುವುದು:

ನಿಮಗೆ ಯಾವುದಾದರೂ ಪ್ರಶ್ನೆಗಳಿದ್ದಲ್ಲಿ, ಅದನ್ನು ನೀವು ಈಗಲೇ ಅಥವಾ ನಂತರ ಅಥವಾ ಅಧ್ಯಯನ ಪ್ರಾರಂಭವಾದ ನಂತರವೂ ಕೇಳಬಹ ುದು. ನಂತರದಲ್ಲಿ ನೀವು ಪ್ರಶ್ನೆಗಳನ್ನು ಕೇಳುವವರಾಗಿದ್ದಲ್ಲಿ, ನೀವು ಸಂಪರ್ಕಿಸಬೇಕಾದವರು ಃ ಕನಕವಲ್ಲಿ ಕೆ. ಕುಂಡೂರಿ, ಸಹಾಯಕ ಪ್ರಾಧ್ಯಾಪಕಿ, ಆರೋಗ್ಯ ಪದ್ಧತಿ ನಿರ್ವಹಣಾ ಅಧ್ಯಯನ ವಿಭಾಗ, ಜಗದ್ಗುರು ಶ್ರೀ ಶಿವರಾತ್ರೀಶ್ವರ ವಿಶ್ವವಿದ್ಯಾನಿಲಯ, ಮೈಸೂರು. ಮೊಬೈಲ್ ಸಂಃ 9980803301, ಮಿಂಚಂಚೆಃ kirankundury@gmail.com, kanakavalli.dhsms@jssuni.edu.in

ಈ ಪ್ರಸ್ತಾವನೆಯನ್ನು ಜೆಎಸ್‌ಎಸ್ ವಿಶ್‌ವಿದ್ಯಾನಿಲಯದ ಎಥಿಕ್ಸ್ ಸಮಿತಿಯು ವಿಮರ್ಶಿಸಿ ಅನಮೋದಿಸಿರುತ್ತದೆ.

ಅಧ್ಯಯನದ ಶೀರ್ಷಿಕೆ ಃ Effectiveness of Self Management in Type2 Diabetic Patients through Ihformation Communication Technology (ICT) Interventions

ವಿಷಯಕಾರರ ಸಹಿ ಃ . .. . **ವಿಷಯಕಾರರ ಹೆಸರುಃ** .. ..

ಹುಟ್ಟಿದ ದಿನಾಂಕ / ವಯಸ್ಸು ಃ .. ..

1	ನಾನು ಈ ಮಾಹಿತಿ ಪುಟದಲ್ಲಿರುವ ಮೇಲೆ ತಿಳಿಸಿರುವ ಅಧ್ಯಯನಕ್ಕೆ ಕುರಿತಂತೆ ಓದಿ ಅರ್ಥೈಸಿಕೊಂಡಿರುತ್ತೇನೆ ಮತ್ತು ಪ್ರಶ್ನೆಗಳನ್ನು ಕೇಳಲು ನನಗೆ ಅವಕಾಶ ಒದಗಿಸಲಾಗಿತ್ತು	( )
2	ಈ ಅಧ್ಯಯನದಲ್ಲಿ ತೊಡಗಿಸಿಕೊಳ್ಳಲು ಇದು ಸ್ವಯಂಪ್ರೇರಿತ ಎಂಬುದನ್ನು ನಾನು ತಿಳಿದಿರುತ್ತೇನೆ ಮತ್ತು ನಾನು ಇದರಿಂದ ಯಾವುದೇ ಸಮಯದಲ್ಲಾದರಲೂ ಯಾವುದೇ ಕಾರಣಗಳನ್ನು ನೀಡದೇ, ಮತ್ತು ನನ್ನ ವೈದ್ಯಕೀಯ ರಕ್ಷಣೆ ಅಥವಾ ಕಾನೂನು ಹಕ್ಕುಗಳಿಗೆ ತೊಂದರೆಯಾಗದ ರೀತಿ ಹಿಂದೆಗೆಯಲು ಸ್ವತಂತ್ರನಾಗಿರುತ್ತೇನೆ	( )
3	ನೈತಿಕ ಸಮಿತಿ ಮತ್ತು ನಿಯಂತ್ರಣಾ ಪ್ರಾಧಿಕಾರವು ನನ್ನ ಆರೋಗ್ಯಕ್ಕೆ ಸಂಬಂಧಿಸಿದಂತಹ ದಾಖಲೆಗಳನ್ನು ಪ್ರಸಕ್ತ ಅಧ್ಯಯನಕ್ಕೆ ಸಂಬಂಧಿಸಿದಂತೆ ಅಥವಾ ಅದಕ್ಕೆ ಸಂಬಂಧಿಸಿದಂತಹ ಸಂಶೋಧನೆಗಳಿಗಾಗಿ ನೋಡಲು ಹಾಗೂ ಒಂದು ಪಕ್ಷ ನಾನು ಇದರಿಂದ ಹಿಂದೆಗೆದಲ್ಲಿ, ನನ್ನ ಅನುಮತಿ ಬೇಕಾಗಿರುವುದಿಲ್ಲವೆಂಬುದನ್ನು ನಾನು ತಿಳಿದಿರುತ್ತೇನೆ. ಅಲ್ಲದೆ, ನನ್ನ ವ್ಯಕ್ತಿಗತ ವಿವರವು ಯಾವುದೇ ತರಹದ ಮಾಹಿತಿಯ ಮೂಲಕ ಯಾವುದೇ ಮೂರನೆಯ ವ್ಯಕ್ತಿಗಳಿಗೆ ಪ್ರಚುರಪಡಿಸುತ್ತಿಲ್ಲ ಎಂಬುದನ್ನು ನಾನು ತಿಳಿದಿರುತ್ತೇನೆ.	( )
4	ಈ ಅಧ್ಯಯನದಿಂದಾಗುವ ಯಾವುದೇ ಮಾಹಿತಿ ಅಥವಾ ಫಲಿತಾಂಶಗಳನ್ನು ಅದು ವೈಜ್ಞಾನಿಕ ಉದ್ದೇಶಕ್ಕಾಗಿ ಎಂಬುದಾಗಿದ್ದು, ಅದರ ಉಪಯೋಗವನ್ನು ನಾನು ನಿರ್ಬಂಧಿಸುವುದಿಲ್ಲ ಎಂದು ಒಪ್ಪಿರುತ್ತೇನೆ.	
5	ಮೇಲಿನ ಅಧ್ಯಯನದಲ್ಲಿ ನಾನು ಭಾಗವಹಿಸಲು ಒಪ್ಪಿರುತ್ತೇನೆ	( )

ವಿಷಯಕಾರರ ಸಹಿ (ಅಥವಾ ಹೆಬ್ಬೆಟ್ಟಿನ ಗುರುತು) ಮತ್ತು ದಿನಾಂಕ

ಸಾಕ್ಷಿಗಳ ಹೆಸರು ಮತ್ತು ಸಹಿ ದಿನಾಂಕದೊಂದಿಗೆ

ಪ್ರಧಾನ ತನಿಖೆದಾರರ ಹೆಸರು ಮತ್ತು ಸಹಿ ದಿನಾಂಕದೊಂದಿಗೆ
