# Supplementary material for: Providing Diabetes Education through Phone Calls Assisted in the Better Control of Hyperglycemia and Improved the Knowledge of Patients on Diabetes Management
Source: Healthcare (Basel). 2023 Feb 10;11(4):528. doi: 10.3390/healthcare11040528 (PMC9957542; doi:10.3390/healthcare11040528)
Supplement: Supplementary file 1 [file healthcare-11-00528-s001.zip › Supplemental information 3-Demographic information and baseline data.pdf]

Patient Profile

Name: ____

Age: ____________ Gender: M ___ F ___

Height: _______ cms; Weight: _______ Kg's; Waist Circumference: ________ BMI: ________

Address: ____

____

Domicile Category: Urban / Semi Urban / Rural

Landline / Mobile for Contact _______________________________

e-mail: ______________________________________

Profession/ occupation: ________________________________

Diabetic since: ________Years

Blood pressure: _____________ Cholesterol: ____________

Do you have any of the following diabetes associated complications?

Eye Problems____ Heart diseases____ Kidney disorders_____ Stroke_____ Diabetic foot_____

Baseline Diabetes management practices

1. How frequently do you visit your doctor?
   a) 3 months
   b) 3-6 months
   c) More than 6 months
2. How frequently you get your blood investigations done?
   a) 3 months
   b) 3-6 months
   c) More than 6 months
3. Blood investigations normally done
   a) Fasting
   b) PPBS
   c) Random
   d) HbA1C
4. What is your HbA1C range?
   a) <6
   b) 6-8
   c) More than 8
5. How frequently do you manage your diet?
   a) Always
   b) Sometimes
   c) Never
6. Do you adhere to medications given by your doctor
   a) Always

b) Sometimes
c) Never

7. What kind of medication form are you in:
    a) Single therapy
    b) Multiple therapy
    c) Insulin

8. How much time do you spend every day on physical activity (walking, jogging, yoga or any fitness program)?
    a) <=15 minutes
    b) 15-30 minutes
    c) >30 minutes

9. How often do you inspect your eyes and foot
    a) Always
    b) Sometimes
    c) Never

10. Are you aware that diabetes can be self- managed?
    a) Yes
    b) No

<u>Baseline Diabetes Knowledge Assessment Questionnaire</u>

1. Symptoms of diabetes include ___________________________________

a) Excessive thirst
b) Hunger
c) Frequent urination
d) Weight loss
e) All of the above

2. Best practices to keep diabetes under control include _____________________

a) Regular exercise
b) Controlled diet
c) Medication adherence
d) Periodic doctor visit
e) HbA1C investigation every 3-4 months
f) All of the above

3. Do alcohol consumption and smoking influences diabetes associated complications
    a) Yes
    b) No

4. Uncontrolled diabetes could lead to these diseases_____________________

a) Heart disease
b) Eye problems
c) Stroke
d) Neurological diseases
e) Kidney diseases
f) Foot ulcers

5. Symptoms of hypo glycaemia includes

a) Dizziness
b) Hunger
c) Sweating
d) Shakiness
e) Anxiety
f) Moodiness

6. How do you manage hypo glycemic conditions?

a) Consume sugar rich food
b) Take rest at least for 15 minutes
c) Consult doctor immediately
d) All of the above

7. Excellent glucose control is indicated by which of the below ranges mentioned

a) <80 MG/DL
b) 80-120 MG/DL
c) 120-180 MG/DL
d) >180 MG/DL

8. Blood test which gives accurate reading of glucose value and has to be done every 3-4 months is ________________________

a) Fasting
b) PPBS
c) HbA1C
