# Supplementary material for: Providing Diabetes Education through Phone Calls Assisted in the Better Control of Hyperglycemia and Improved the Knowledge of Patients on Diabetes Management
Source: Healthcare (Basel). 2023 Feb 10;11(4):528. doi: 10.3390/healthcare11040528 (PMC9957542; doi:10.3390/healthcare11040528)
Supplement: Supplementary file 1 [file healthcare-11-00528-s001.zip › Supplemental information 6.1- Calling Script Kannada.pdf]

Calling Script -Kannada (Local Language)

ಶುಭೋದಯ / ಮಧ್ಯಾಹ್ನ / ಸಂಜೆ

ನಾವು "ಮಧುಮೇಹಾ ನಿರ್ವಾಹನೆ" ("ಮಧುಮೇಹ ನಿರ್ವಹಣಾ ಕಾರ್ಯಕ್ರಮ") ದಿಂದ ಕರೆಯುತ್ತಿದ್ದೇವೆ, ನಾನು ಈಗ ನಿಮ್ಮೊಂದಿಗೆ ಮಾತನಾಡಬಹುದೇ?

"ಹೌದು" ಆಗಿದ್ದರೆ ಮುಂದುವರಿಸಿ, "ಇಲ್ಲ" ಆಗಿದ್ದರೆ ಅವರೊಂದಿಗೆ ಮಾತನಾಡಲು ಉತ್ತಮ ಸಮಯವನ್ನು ಕೇಳಿ, ಧನ್ಯವಾದಗಳು ಎಂದು ಹೇಳಿ

ದೃಷ್ಟಿಕೋನ ತರಗತಿಯಲ್ಲಿ ಹೇಳಿದಂತೆ, ಮಧುಮೇಹ ಸ್ವ-ನಿರ್ವಹಣೆಯ ಕುರಿತು ಕೆಲವು ಮಾಹಿತಿಯನ್ನು ಹಂಚಿಕೊಳ್ಳಲು ನಾವು ಕರೆ ಮಾಡಿದ್ದೇವೆ

ಮಧುಮೇಹವು ದೀರ್ಘಕಾಲದ ಕಾಯಿಲೆಯಾಗಿದ್ದು, ಇದು ಜೀವನ ಶೈಲಿಯ ನಡವಳಿಕೆಗಳಿಂದ ಉಂಟಾಗುತ್ತದೆ, ಕೆಲವೊಮ್ಮೆ ಪೋಷಕರಿಂದ ಆನುವಂಶಿಕವಾಗಿ ಕಂಡುಬರುತ್ತದೆ. ರಕ್ತದಲ್ಲಿನ ಸಕ್ಕರೆಯನ್ನು ನಿಯಂತ್ರಿಸಲು ನಮ್ಮ ದೇಹದಲ್ಲಿ ಸಾಕಷ್ಟು ಇನ್ಸುಲಿನ್ ಇಲ್ಲದಿದ್ದಾಗ ಮಧುಮೇಹವು ಮೂಲತಃ ಸಂಭವಿಸುತ್ತದೆ. ನಾವು ಪ್ರತಿ ಬಾರಿಯೂ ಏನನ್ನು ಸೇವಿಸುತ್ತೇವೆಯೋ ಅದು ಸಕ್ಕರೆಯಾಗಿ ಪರಿವರ್ತನೆಗೊಳ್ಳುತ್ತದೆ, ಅದು ನಮ್ಮ ಕೆಲಸವನ್ನು ಮಾಡಲು ಶಕ್ತಿಯನ್ನು ನೀಡುತ್ತದೆ. ನಮ್ಮ ದೇಹದಲ್ಲಿ ಇನ್ಸುಲಿನ್ ಎಂಬ ಹಾರ್ಮೋನ್ ಇದೆ, ಇದು ರಕ್ತದಲ್ಲಿನ ಸಕ್ಕರೆ ಮಟ್ಟವನ್ನು ಹೆಚ್ಚು ಮತ್ತು ಕಡಿಮೆ ಆಗದಂತೆ ನಿಯಂತ್ರಿಸುತ್ತದೆ. ದೇಹದಲ್ಲಿ ಹೆಚ್ಚು ಸಕ್ಕರೆ ಇದ್ದಾಗ, ಈ ಹಾರ್ಮೋನ್ ರಕ್ತದಲ್ಲಿನ ಸಕ್ಕರೆಯನ್ನು ನಿಯಂತ್ರಿಸಲು ವಿಫಲವಾಗುತ್ತದೆ, ಅಲ್ಲಿ ರಕ್ತದಲ್ಲಿನ ಸಕ್ಕರೆಯನ್ನು ಹೆಚ್ಚಿಸಿ ಮಧುಮೇಹಕ್ಕೆ ಕಾರಣವಾಗುತ್ತದೆ.

ನಿಯಂತ್ರಿತ ಆಹಾರ ಸೇವನೆ, ದೈಹಿಕ ವ್ಯಾಯಾಮ, ation ಷಧಿಗಳ ಅನುಸರಣೆ ಮತ್ತು ಆವರ್ತಕ ವೈದ್ಯರ ಭೇಟಿಯಂತಹ ಜೀವನ ಶೈಲಿಯ ನಡವಳಿಕೆಯ ಮಾರ್ಪಾಡುಗಳ ಮೂಲಕ ಮಧುಮೇಹವನ್ನು ಹೆಚ್ಚಿನ ಪ್ರಮಾಣದಲ್ಲಿ ನಿರ್ವಹಿಸಬಹುದು.

ಆಹಾರ:

· ಮಧುಮೇಹ ರೋಗಿಗಳು ಸಕ್ಕರೆ ಭರಿತ ಆಹಾರಗಳಾದ ಸಿಹಿತಿಂಡಿಗಳು, ಬೇಕರಿ ವಸ್ತುಗಳು, ತಂಪಾದ ಪಾನೀಯಗಳು ಮತ್ತು ತ್ವರಿತ ಆಹಾರಗಳನ್ನು ತಪ್ಪಿಸಬೇಕು. ನಿಯತಕಾಲಿಕವಾಗಿ ಸೀಮಿತ ಪ್ರಮಾಣದಲ್ಲಿ ತಿನ್ನುತ್ತಿರಬೇಕು. ನಾವು ಏಕಕಾಲದಲ್ಲಿ ಹೆಚ್ಚು ಆಹಾರವನ್ನು ಸೇವಿಸಿದರೆ ಅದು ರಕ್ತದಲ್ಲಿನ ಸಕ್ಕರೆ ಮಟ್ಟವನ್ನು ಹೆಚ್ಚಿಸುತ್ತದೆ. ನಿಯತಕಾಲಿಕವಾಗಿ ಸೀಮಿತ ಪ್ರಮಾಣದಲ್ಲಿ ತಿನ್ನುವುದು ರಕ್ತದಲ್ಲಿನ ಸಕ್ಕರೆ ಮಟ್ಟವನ್ನು ಸಾಮಾನ್ಯವಾಗಿಸಲು ಸಹಾಯ ಮಾಡುತ್ತದೆ

ರಾಗಿ, ಗೋಧಿ, ಕಚ್ಚಾ ತರಕಾರಿಗಳು (ಕ್ಯಾರೆಟ್, ಸೌತೆಕಾಯಿ, ಎಲೆಕೋಸು ಇತ್ಯಾದಿ) ನಂತಹ ನಾರಿನಂಶವಿರುವ ಆಹಾರವನ್ನು ಸೇವಿಸಬೇಕು

ಹಣ್ಣುಗಳು ಸಕ್ಕರೆ ಅಂಶದಿಂದ ಸಮೃದ್ಧವಾಗಿರುವುದರಿಂದ ಸೀಮಿತ ಪ್ರಮಾಣದಲ್ಲಿ ಸೇವಿಸಬೇಕು

ಆಹಾರ ಸೇವನೆಯ ಬಗ್ಗೆ ನಿಮಗೆ ಯಾವುದೇ ಅನುಮಾನಗಳಿದ್ದರೆ ದಯವಿಟ್ಟು ನಿಮ್ಮ ಆಹಾರ ತಜ್ಞರನ್ನು ಸಂಪರ್ಕಿಸಿ.

ದೈಹಿಕ ಚಟುವಟಿಕೆ: ರಕ್ತದಲ್ಲಿನ ಸಕ್ಕರೆ ಮಟ್ಟವನ್ನು ನಿಯಂತ್ರಿಸಲು ವ್ಯಾಯಾಮ ಸಾಬೀತಾಗಿದೆ. ನೀವು ವ್ಯಾಯಾಮ ಮಾಡುವಾಗ, ನಮ್ಮ ದೇಹದಲ್ಲಿನ ಸ್ನಾಯುಗಳು ಹೆಚ್ಚುವರಿ ರಕ್ತದಲ್ಲಿನ ಸಕ್ಕರೆಯನ್ನು ಬಳಸುತ್ತವೆ, ಅಲ್ಲಿ ರಕ್ತದಲ್ಲಿನ ಸಕ್ಕರೆ ಮೌಲ್ಯಗಳನ್ನು ಸಾಮಾನ್ಯ ಮಟ್ಟಕ್ಕೆ ಇಳಿಸುತ್ತದೆ

· ವಾಕಿಂಗ್, ಸೈಕ್ಲಿಂಗ್, ಓಟ, ಜಾಗಿಂಗ್, ಈಜು ದೈನಂದಿನ ಆಧಾರದ ಮೇಲೆ ಪರಿಗಣಿಸಬಹುದಾದ ದೈಹಿಕ ಚಟುವಟಿಕೆಗಳಲ್ಲಿ ಕೆಲವು

ನಿಯಮಿತವಾಗಿ ಮನೆ ಹಿಡಿದಿಟ್ಟುಕೊಳ್ಳುವ ಕೆಲಸವನ್ನು ವ್ಯಾಯಾಮ ಎಂದು ಪರಿಗಣಿಸಲಾಗುವುದಿಲ್ಲ. ಹವಾಮಾನ ಅಥವಾ ಯಾವುದೇ ಕುಟುಂಬದ ಸಂದರ್ಭಗಳು ವ್ಯಾಯಾಮ ಮಾಡಲು ಅನುಮತಿಸದಿದ್ದರೆ, ಮೆಟ್ಟಿಲುಗಳನ್ನು ಮೇಲಕ್ಕೆ ಮತ್ತು ಕೆಳಕ್ಕೆ ಏರಿಸುವುದನ್ನು ಸಹ ಮಾಡಬಹುದು

ಎಲ್ಲಾ ರೀತಿಯ ವ್ಯಾಯಾಮಗಳಲ್ಲಿ, ವಾಕಿಂಗ್ ವ್ಯಾಯಾಮದ ಅತ್ಯುತ್ತಮ ರೂಪವೆಂದು ಸಾಬೀತಾಗಿದೆ. ಚುರುಕಾದ ವಾಕಿಂಗ್ (ಉದ್ದದ ಕಾಲು ವ್ಯಾಪ್ತಿಯೊಂದಿಗೆ ನಿಮಿಷಕ್ಕೆ 100 ಹೆಜ್ಜೆಗಳು) ರಕ್ತದಲ್ಲಿನ ಸಕ್ಕರೆ ಮೌಲ್ಯವನ್ನು ಕಡಿಮೆ ಮಾಡುತ್ತದೆ ಮತ್ತು ಇನ್ಸುಲಿನ್ ಉತ್ಪಾದನೆಯನ್ನು ಹೆಚ್ಚಿಸುತ್ತದೆ ಎಂದು ಸಾಬೀತಾಗಿದೆ

ದಿನಕ್ಕೆ ಕನಿಷ್ಠ 30 ನಿಮಿಷಗಳ ವ್ಯಾಯಾಮ ರಕ್ತದಲ್ಲಿನ ಸಕ್ಕರೆಯನ್ನು ನಿಯಂತ್ರಣದಲ್ಲಿಡಲು ಸಹಾಯ ಮಾಡುತ್ತದೆ

ವ್ಯಾಯಾಮ ಯೋಜನೆ ಅಥವಾ ಇನ್ನಾವುದೇ ಸ್ಪಷ್ಟೀಕರಣಗಳಲ್ಲಿ ನಿಮಗೆ ಸಹಾಯ ಬೇಕಾದರೆ ದಯವಿಟ್ಟು ನಿಮ್ಮ ವೈದ್ಯರನ್ನು ಸಂಪರ್ಕಿಸಿ

- ಔಷಧಿಗಳ ಅನುಸರಣೆ:

· ದೇಹದಲ್ಲಿನ ರಕ್ತದಲ್ಲಿನ ಸಕ್ಕರೆ ಮಟ್ಟವನ್ನು ನಿಯಂತ್ರಿಸಲು ins ಷಧಿಗಳು ಮತ್ತು ಇನ್ಸುಲಿನ್ ಹೆಚ್ಚುವರಿ ಇನ್ಸುಲಿನ್ ಉತ್ಪಾದಿಸಲು ಸಹಾಯ ಮಾಡುತ್ತದೆ

ಡೋಸೇಜ್ ಅನ್ನು ಕಳೆದುಕೊಳ್ಳದೆ ವೈದ್ಯರ ನಿರ್ದೇಶನದಂತೆ ines ಷಧಿಗಳನ್ನು ತೆಗೆದುಕೊಳ್ಳಬೇಕು

ತಪ್ಪಿದ ಡೋಸೇಜ್‌ಗಳನ್ನು ತಪ್ಪಿಸಲು ಔಷಧಿಗಳನ್ನು ಸಮಯಕ್ಕಿಂತ ಒಂದು ವಾರ ಮುಂಚಿತವಾಗಿ ಮನೆಯಲ್ಲಿ ಸಂಗ್ರಹಿಸಬೇಕು

ಕೆಲವೊಮ್ಮೆ, ರಕ್ತದಲ್ಲಿನ ಸಕ್ಕರೆ ಮಟ್ಟವು ಇದ್ದಕ್ಕಿದ್ದಂತೆ ಕಡಿಮೆಯಾದರೆ, ಸಕ್ಕರೆ ಸಮೃದ್ಧವಾಗಿರುವ ಆಹಾರವನ್ನು ತಕ್ಷಣ ಸೇವಿಸಿ, ಸ್ವಲ್ಪ ವಿಶ್ರಾಂತಿ ತೆಗೆದುಕೊಂಡು ತಕ್ಷಣ ವೈದ್ಯರನ್ನು ಸಂಪರ್ಕಿಸಿ

· ಯಾವುದೇ ಕಾರಣಕ್ಕೂ ಷಧಿಗಳನ್ನು ತಪ್ಪಿಸಬಾರದು

ಔಷಧಿಗಳ ಬಗ್ಗೆ ಯಾವುದೇ ಸಮಸ್ಯೆಗಳು ಅಥವಾ ಅನುಮಾನಗಳು ಅಥವಾ ಇನ್ಸುಲಿನ್ ನೀಡುವುದು ದಯವಿಟ್ಟು ನಿಮ್ಮ ವೈದ್ಯರನ್ನು ಸಂಪರ್ಕಿಸಿ

ಜೀವನ ಶೈಲಿಯ ನಡವಳಿಕೆಗಳು:

ಆಲ್ಕೋಹಾಲ್ ಸೇವನೆಯು ರಕ್ತದಲ್ಲಿನ ಸಕ್ಕರೆ ಮಟ್ಟವನ್ನು ಹೆಚ್ಚಿಸುತ್ತದೆ. ಆದ್ದರಿಂದ ಆಲ್ಕೊಹಾಲ್ ಸೇವನೆಯನ್ನು ತಪ್ಪಿಸಬೇಕು

ಸಮಯೋಚಿತ ಆಹಾರ ಸೇವನೆಯಂತಹ ಆರೋಗ್ಯಕರ ದಿನಚರಿಯನ್ನು ಅಳವಡಿಸಿಕೊಳ್ಳುವುದು ಮತ್ತು ಸಾಕಷ್ಟು ವಿಶ್ರಾಂತಿ ಪಡೆಯುವುದು ರಕ್ತದಲ್ಲಿನ ಸಕ್ಕರೆ ಮಟ್ಟವನ್ನು ನಿರ್ವಹಿಸಲು ಸಹಾಯ ಮಾಡುತ್ತದೆ

· ಒತ್ತಡ ಮತ್ತು ಆತಂಕವು ರಕ್ತದಲ್ಲಿನ ಸಕ್ಕರೆ ಮಟ್ಟವನ್ನು ಹೆಚ್ಚಿಸುತ್ತದೆ. ನೀವು ಬೇಸರಗೊಂಡಿದ್ದರೆ ಅಥವಾ ಏಕಾಂಗಿಯಾಗಿರುತ್ತಿದ್ದರೆ, ಆತಂಕ ಮತ್ತು ಒತ್ತಡವನ್ನು ನಿಭಾಯಿಸಲು, ಹೊಲಿಗೆ, ಚಿತ್ರಕಲೆ, ಪುಸ್ತಕ ಓದುವಿಕೆ, ದೇವಾಲಯಗಳಿಗೆ ಭೇಟಿ ನೀಡುವುದು ಅಥವಾ ಪೂಜೆ ಮಾಡುವುದು ಮುಂತಾದ ಕೆಲವು ನೆಚ್ಚಿನ ಚಟುವಟಿಕೆಗಳಲ್ಲಿ ನಿಮ್ಮನ್ನು

ತೊಡಗಿಸಿಕೊಳ್ಳಿ. ಈ ಚಟುವಟಿಕೆಗಳು ನಿಮ್ಮ ಮನಸ್ಸನ್ನು ಶಾಂತವಾಗಿ ಮತ್ತು ಶಾಂತವಾಗಿಡಲು ಸಹಾಯ ಮಾಡುತ್ತದೆ, ಇದರಿಂದಾಗಿ ನಿಮ್ಮ ರಕ್ತದಲ್ಲಿನ ಗ್ಲೂಕೋಸ್ ಮಟ್ಟಕ್ಕೆ ಸಹಾಯ ಮಾಡುತ್ತದೆ

ನಿಮ್ಮ ಸುತ್ತಮುತ್ತಲಿನ ಜನರೊಂದಿಗೆ ವಾದಗಳನ್ನು ತಪ್ಪಿಸಲು ಸಾಧ್ಯವಾದಷ್ಟು, ಪ್ರತಿ ಸನ್ನಿವೇಶದಲ್ಲೂ ಶಾಂತವಾಗಿರಿ ಮತ್ತು ವಿಶ್ರಾಂತಿ ಪಡೆಯಿರಿ. ಏಕೆಂದರೆ ಆತಂಕ ಮತ್ತು ಕೋಪವು ನಿಮ್ಮ ರಕ್ತದಲ್ಲಿನ ಗ್ಲೂಕೋಸ್ ಮೌಲ್ಯಗಳನ್ನು ಹೆಚ್ಚಿಸುತ್ತದೆ.

ಅನಿಯಂತ್ರಿತ ಮಧುಮೇಹ:

ಆಹಾರ ನಿಯಂತ್ರಣ, ನಿಯಮಿತ ದೈಹಿಕ ವ್ಯಾಯಾಮ ಮತ್ತು ation ಷಧಿಗಳ ಅನುಸರಣೆ ಮತ್ತು ಆರೋಗ್ಯಕರ ಜೀವನ ಶೈಲಿಯ ನಡವಳಿಕೆಗಳನ್ನು ಅಳವಡಿಸಿಕೊಳ್ಳುವ ಮೂಲಕ ಮಧುಮೇಹವನ್ನು ಪರಿಣಾಮಕಾರಿಯಾಗಿ ನಿರ್ವಹಿಸಬಹುದು.

ಸರಿಯಾದ ಕಾಳಜಿಯನ್ನು ತೆಗೆದುಕೊಳ್ಳದಿದ್ದರೆ, ಮಧುಮೇಹ ಅನಿಯಂತ್ರಿತವಾಗಿದ್ದರೆ, ಕಣ್ಣಿನ ಕಾಯಿಲೆಗಳು, ಹೃದ್ರೋಗಗಳು, ಮೂತ್ರಪಿಂಡಗಳ ವೈಫಲ್ಯ, ಚರ್ಮ ರೋಗಗಳು, ಕಾಲು ಹುಣ್ಣುಗಳು, ನರವೈಜ್ಞಾನಿಕ ಕಾಯಿಲೆಗಳು ಮತ್ತು ಪಾರ್ಶ್ವವಾಯು ಮುಂತಾದ ವಿವಿಧ ಕಾಯಿಲೆಗಳಿಗೆ ಕಾರಣವಾಗುತ್ತದೆ

ಯಾವುದೇ ಸಮಸ್ಯೆಗಳನ್ನು ಗಮನಿಸಿದರೆ, ನೀವು ತಕ್ಷಣ ವೈದ್ಯರನ್ನು ಸಂಪರ್ಕಿಸಬೇಕು

ಮಧುಮೇಹ ತನಿಖೆ:

ನಿಮ್ಮ ರಕ್ತದಲ್ಲಿನ ಗ್ಲೂಕೋಸ್ ಮೌಲ್ಯಗಳನ್ನು ಪತ್ತೆಹಚ್ಚಲು ಆವರ್ತಕ ತನಿಖೆಗಳು ಅತ್ಯಗತ್ಯ

ಈ ರಕ್ತದಲ್ಲಿನ ಸಕ್ಕರೆ ತನಿಖೆಗಳು ಉಪವಾಸ, ಪಿಪಿಬಿಎಸ್ (ಆಹಾರದ ನಂತರ 1.5 ರಿಂದ 2 ಗಂಟೆಗಳ ನಂತರ) ಅಥವಾ ಯಾದೃಚ್ ಗ್ಲೂಕೋಸ್ ಪರೀಕ್ಷೆ ಆಗಿರಬಹುದು, ಇದನ್ನು ಯಾವುದೇ ಸಮಯದಲ್ಲಿ ಮಾಡಬಹುದು. ಸಕ್ಕರೆ ಮೌಲ್ಯಗಳ ಉತ್ತಮ ನಿಯಂತ್ರಣ 80-120 ಮಿಗ್ರಾಂ / ಡಿಎಲ್ ವರೆಗೆ ಇರುತ್ತದೆ. ಭೇಟಿಗಳ ಸಮಯದಲ್ಲಿ ವೈದ್ಯರಿಗೆ ತೋರಿಸಲು ದಯವಿಟ್ಟು ನಿಮ್ಮ ವಾಚನಗೋಷ್ಠಿಯನ್ನು ರೆಕಾರ್ಡ್ ಮಾಡಿ ಅಥವಾ ನಿಮ್ಮ ವರದಿಗಳನ್ನು ಉಳಿಸಿ

ಈ ತನಿಖೆಗಳು ನಿಮ್ಮ ದೇಹದಲ್ಲಿ ನಿರ್ದಿಷ್ಟ ದಿನ ಅಥವಾ ಸಮಯದಲ್ಲಿ ರಕ್ತದಲ್ಲಿನ ಗ್ಲೂಕೋಸ್ ಮೌಲ್ಯಗಳನ್ನು ನೀಡುತ್ತದೆ

· ಆದಾಗ್ಯೂ, ಎಚ್‌ಬಿಎ 1 ಸಿ ತನಿಖೆಗಳು ನಿಮ್ಮ ರಕ್ತದಲ್ಲಿನ ಗ್ಲೂಕೋಸ್‌ನ ಸರಾಸರಿ ಮೌಲ್ಯವನ್ನು 3-4 ತಿಂಗಳುಗಳವರೆಗೆ ನೀಡುತ್ತದೆ. ಈ ಪರೀಕ್ಷೆಯನ್ನು ಪ್ರತಿ 3-4 ತಿಂಗಳಿಗೊಮ್ಮೆ ಪುನರಾವರ್ತಿಸಬೇಕಾಗಿದೆ, ಇದು ನಿಮ್ಮ ಸಕ್ಕರೆ ಮೌಲ್ಯಗಳನ್ನು ನಿಯಂತ್ರಣದಲ್ಲಿಡಲು ಅಗತ್ಯವಿರುವ ಔಷಧಿಗಳು, ವ್ಯಾಯಾಮ ಯೋಜನೆ ಮತ್ತು ಯಾವುದೇ ಜೀವನ ಶೈಲಿಯ ನಡವಳಿಕೆಯ ಬದಲಾವಣೆಗಳನ್ನು ಶಿಫಾರಸು ಮಾಡಲು ನಮಗೆ ಮತ್ತು ವೈದ್ಯರಿಗೆ ಸಹಾಯ ಮಾಡುತ್ತದೆ. ರಕ್ತದಲ್ಲಿನ ಸಕ್ಕರೆಯ ಉತ್ತಮ ನಿಯಂತ್ರಣಕ್ಕಾಗಿ ಎಚ್‌ಬಿಎ 1 ಸಿ ಮೌಲ್ಯವು 6-7% ಆಗಿರಬೇಕು.

ಆವರ್ತಕ ವೈದ್ಯರ ಭೇಟಿ:

ರಕ್ತದಲ್ಲಿನ ಸಕ್ಕರೆ ಮಟ್ಟ ಅಥವಾ ಇತರ ಯಾವುದೇ ಮಧುಮೇಹ ಸಂಬಂಧಿತ ತೊಂದರೆಗಳನ್ನು ಪರೀಕ್ಷಿಸಲು ವೈದ್ಯರನ್ನು ತಿಂಗಳಿಗೆ ಒಮ್ಮೆಯಾದರೂ ಸಂಪರ್ಕಿಸಬೇಕು

ಅಗತ್ಯವಿರುವ ಯಾವುದೇ ಅನುಮಾನಗಳು ಅಥವಾ ಸ್ಪಷ್ಟೀಕರಣಗಳನ್ನು ಪ್ರತಿ ಭೇಟಿಯ ಸಮಯದಲ್ಲಿ ವೈದ್ಯರೊಂದಿಗೆ ಚರ್ಚಿಸಬಹುದು

ನಿರಂತರ ಮಧುಮೇಹ ಶಿಕ್ಷಣವನ್ನು ನೀಡುವ ಮೂಲಕ ಮಧುಮೇಹದ ಸ್ವಯಂ ನಿರ್ವಹಣೆಯಲ್ಲಿ ಭಾಗವಹಿಸುವವರನ್ನು ಪ್ರೋತ್ಸಾಹಿಸುವ ಮತ್ತು ಪ್ರೇರೇಪಿಸುವ ಉದ್ದೇಶವನ್ನು ಈ ಅಧ್ಯಯನ ಹೊಂದಿದೆ. ಇದು ಮಧುಮೇಹ ರೋಗಿಗಳಿಗೆ ಅವರ ರೋಗ ನಿರ್ವಹಣೆಯಲ್ಲಿ ಕೈ ಹಿಡಿಯುವಂತೆ ಕಾರ್ಯನಿರ್ವಹಿಸುತ್ತದೆ ಆದರೆ ವೈದ್ಯರ ಸಲಹೆಯನ್ನು ಬದಲಿಸುವ ಉದ್ದೇಶವನ್ನು ಹೊಂದಿಲ್ಲ. ದಯವಿಟ್ಟು ನಿಮ್ಮ ವೈದ್ಯರ ಸೂಚನೆಗಳನ್ನು ಅನುಸರಿಸಿ ಮತ್ತು ಅದಕ್ಕೆ ಬದ್ಧರಾಗಿರಿ. ಧನ್ಯವಾದಗಳು.

ವಾರ 1: ಭಾಗವಹಿಸುವವರಿಗೆ ಸಂಪೂರ್ಣ ವಿಷಯವನ್ನು ತಲುಪಿಸಿ. ಮಧ್ಯದಲ್ಲಿ ವಿರಾಮಗೊಳಿಸಿ ಮತ್ತು ಅವರು ನಿಮ್ಮಲ್ಲಿ ಸಕ್ರಿಯವಾಗಿ ಕೇಳುತ್ತಾರೆಯೇ ಎಂದು ಪರಿಶೀಲಿಸಲು ಟೋನ್ ವ್ಯತ್ಯಾಸಗಳನ್ನು ತೋರಿಸಿ.

ವಾರ 2: ವಾರ 1 ರ ಸಮಯದಲ್ಲಿ ವಿವರವಾದ ವಿಷಯ ವಿತರಣೆಯು ಸಂಭವಿಸಿರುವುದರಿಂದ, ಭಾಗವಹಿಸುವವರು ಕೇಳುವಲ್ಲಿ ಆಸಕ್ತಿ ತೋರಿಸಿದರೆ ಈ ಪ್ರತಿಯೊಂದು ಘಟಕಗಳ ಬಗ್ಗೆ ವಿವರವಾದ ಮಾಹಿತಿಯನ್ನು ನೀಡಿ. ಇಲ್ಲದಿದ್ದರೆ ಈ ಎಲ್ಲಾ ನಿಯತಾಂಕಗಳಲ್ಲಿ ಅವರು ಹೇಗೆ ಕಾರ್ಯನಿರ್ವಹಿಸುತ್ತಿದ್ದಾರೆ ಎಂಬುದನ್ನು ಪರಿಶೀಲಿಸಿ ಮತ್ತು ದಪ್ಪ ವಿಷಯವನ್ನು ತಲುಪಿಸಿ.

ವಾರ 3: ವಿವರವಾದ ವಿಷಯ ವಿತರಣೆಯು ವಾರ 1 ರ ಸಮಯದಲ್ಲಿ ಸಂಭವಿಸಿದ್ದರಿಂದ, ಭಾಗವಹಿಸುವವರು ಕೇಳುವಲ್ಲಿ ಆಸಕ್ತಿ ತೋರಿಸಿದರೆ ಈ ಪ್ರತಿಯೊಂದು ಘಟಕಗಳ ಬಗ್ಗೆ ವಿವರವಾದ ಮಾಹಿತಿಯನ್ನು ನೀಡಿ. ಇಲ್ಲದಿದ್ದರೆ ಈ ಎಲ್ಲಾ ನಿಯತಾಂಕಗಳಲ್ಲಿ ಅವರು ಹೇಗೆ ಕಾರ್ಯನಿರ್ವಹಿಸುತ್ತಿದ್ದಾರೆ ಎಂಬುದನ್ನು ಪರಿಶೀಲಿಸಿ ಮತ್ತು ದಪ್ಪ ವಿಷಯವನ್ನು ತಲುಪಿಸಿ.

ವಾರ 4: ಭಾಗವಹಿಸುವವರು ತಮ್ಮ ರೋಗ ನಿರ್ವಹಣಾ ಅಭ್ಯಾಸಗಳನ್ನು ಪರಿಶೀಲಿಸುವ ಮೂಲಕ ಈ ಎಲ್ಲಾ ವಾರಗಳಿಂದ ಪಡೆದ ಮಧುಮೇಹ ಜ್ಞಾನದ ಮೌಲ್ಯಮಾಪನ
