# Supplementary material for: Providing Diabetes Education through Phone Calls Assisted in the Better Control of Hyperglycemia and Improved the Knowledge of Patients on Diabetes Management
Source: Healthcare (Basel). 2023 Feb 10;11(4):528. doi: 10.3390/healthcare11040528 (PMC9957542; doi:10.3390/healthcare11040528)
Supplement: Supplementary file 1 [file healthcare-11-00528-s001.zip › Supplemental information 6.2- Calling Script English.pdf]

Call Script Self-Management of Diabetes

Good morning/ afternoon/ evening

We are calling from "Madhumeha Nirvahane"(" Diabetes Management Program"), Can I speak to you now?

If "Yes" then continue, If "No" then ask the best time to talk to them, Say thank you

As mentioned in the orientation class, **we had called to share some information on diabetes self-management**

Diabetes is a chronic disease which occurs due to life style behaviors, sometimes as hereditary from parents. Diabetes basically occurs when there is no enough insulin in our body to control blood sugar. Whatever we consume every time, that gets converted into sugar, which gives us energy to do our work. There is a hormone called insulin in our body, that regulates the blood sugar level from getting too high and too low. When there is too much sugar in the body, this hormone fails to control the sugar in the blood, there by increasing blood sugar and leading to diabetes.

Diabetes can be managed to a great extent through life style behavior modifications such as eating controlled diet, physical exercise, medication adherence and periodic doctor visit

Diet:

- **Diabetic patients should avoid sugar rich foods** such as sweets, bakery items, cool drinks and fast foods
- **Should be eating in limited quantities periodically**. If we consume too much food at once it increases blood sugar level. Eating periodically in limited quantities would help to keep the blood sugar levels normal through out
- Should consume foods rich in fiber content such as ragi, wheat, raw vegetables (carrot, cucumber, cabbage etc)
- As fruits are rich in sugar content, limited amount should be consumed
- Please consult your dietician if you have any doubts on diet intake.

Physical activity:

- **Exercise is proven to control blood sugar level.** When you exercise, muscles in our body uses the extra blood sugar, there by reducing blood sugar values to normal levels
- Walking, cycling, running, jogging, swimming are few of the physical activities that can be considered on daily basis
- Regular house hold work cannot be considered as exercise. If weather or any family situations did not permit to do exercise, climbing up and down the steps could also be done

- Among all the types of exercise, **walking is proven to be the best form of exercise**. Brisk walking (100 steps per minutes with long foot spans) is proven to decrease blood sugar value and increase insulin production
- **Minimum of 30 minutes of exercise a day helps to keep blood sugar under control**
- **Please consult your physician if you need assistance in exercise plan or any other clarifications**

Medication adherence:

- Medicines and insulin helps in generating extra insulin to control the blood sugar levels in the body
- **Medicines should be taken as directed by doctor without missing a single dosage**
- Medicines should be stocked at home a week ahead of time to avoid missed dosages
- If sometimes, blood sugar level drops down suddenly, consume sugary rich food instantly, take some rest and contact doctor immediately
- **Medicines should not be missed at any reason**
- **Any issues or doubts about medications or administering insulin please contact your physician**

Life style behaviors:

- **Consumption of alcohol increases blood sugar levels. So alcohol consumption should be avoided**
- Adopting healthy routine such as **timely food consumption** and getting **adequate rest** helps in managing blood sugar levels
- Stress and anxiety also increase blood sugar levels. If you are bored or staying alone, to cope up with anxiety and stress, engage yourself in some favorite activities such as stitching, painting, book reading, visiting temples or doing pooja. These activities will help you to **keep your mind calm and relaxed**, thereby helping in your blood glucose levels
- As much possible avoid arguments with people around you, be calm and relaxed in every situation. Because **anxiety and anger can elevate your blood glucose values.**

Uncontrolled diabetes:

- Diabetes can be managed effectively by adopting diet control, regular physical exercise and medication adherence and healthy life style behaviors.
- If proper care is not taken, **diabetes if uncontrolled, leads to various other diseases such as eye disorders, heart diseases, failure of kidneys, skin diseases, foot ulcers, neurological diseases and stroke**
- **If any of the problems are observed, you have to contact doctor immediately**

Diabetes investigations:

- Periodic investigations are vital to track your blood glucose values

- These blood sugar investigations could be fasting, PPBS (1.5 to 2 hours after food) or random glucose testing which can be done any time. Good control of sugar values ranges from 80-120 mg/dl. Please record your readings or save your reports to show it to doctor during visits
- These investigations give blood glucose values in your body on that particular day or time
- However, **HbA1C investigations gives the average value of your blood glucose for 3-4 months. This test has to be repeated for every 3-4 months**, which helps us and the doctors to decide on prescribing medications, meal plan, exercise plan and any life style behavior changes required to keep your sugar values under control. **HbA1C value should be 6-7% for good control of blood sugar.**

Periodic doctor visit:

- **Doctor should be consulted at least once in a month, to get checked for blood sugar levels or any other diabetes associated complications**
- **Any doubts or clarifications required can be discussed with doctor during each visit**

The study is aimed at encouraging and motivating the participants in self-management of diabetes by providing continuous diabetes education. This acts as a hand holding to diabetic patients in their disease management but not intended to substitute doctor's advice. Kindly follow your doctor's instructions and adhere to the same. Thank you.

Week1: Deliver the entire content to the participant. Pause in between and show tone variations to check if they are actively listening to you.

Week2: Since detailed content delivery had happened during Week1, give detail information on each of these components if the participant shows interest in hearing. Or else check how they are doing in all these parameters and deliver the bolded content.

Week3: Since detailed content delivery had happened during Week1, give detail information on each of these components if the participant shows interest in hearing. Or else check how they are doing in all these parameters and deliver the bolded content.

Week4: Assessment of Diabetes knowledge acquired by the participant from all these weeks by checking their disease management practices
