# Supplementary material for: Providing Diabetes Education through Phone Calls Assisted in the Better Control of Hyperglycemia and Improved the Knowledge of Patients on Diabetes Management
Source: Healthcare (Basel). 2023 Feb 10;11(4):528. doi: 10.3390/healthcare11040528 (PMC9957542; doi:10.3390/healthcare11040528)
Supplement: Supplementary file 1 [file healthcare-11-00528-s001.zip › Supplemental information 7 - Weeekly phonecall log sheet.pdf]

Weekly phone call log sheet

Diabetes Education

Diabetes Self-Management Practices					
Introduction on diabetes	Diet	Exercise	Medication adherence	Periodic doctor visits	Blood glucose investigations (Fasting, PPBS and HbA1C

Life style behaviours				Uncontrolled Diabetes						
Alcohol consumption	Timely food consumption	Stress & anxiety	Family situations	Eye problem	Kidney Disorders	Cardiac Diseases	Stroke	Skin diseases	Foot Ulcers	Additional remarks
