# Supplementary material for: Providing Diabetes Education through Phone Calls Assisted in the Better Control of Hyperglycemia and Improved the Knowledge of Patients on Diabetes Management
Source: Healthcare (Basel). 2023 Feb 10;11(4):528. doi: 10.3390/healthcare11040528 (PMC9957542; doi:10.3390/healthcare11040528)
Supplement: Supplementary file 1 [file healthcare-11-00528-s001.zip › Supplemental information 8 - Data sheet for capturing diabetes management practices.pdf]

Data Sheet for capturing diabetes management practices of participants

Patient ID:

Madhumeha Nirvahane (Diabetes Management)												
Call #	Date	Call Duration		Dietary Habits	Physical Exercise		Medication Adherence	Glucose Tests		Eye & Foot care	Doctor's visits	Remarks
		Start time	End		Exercise	Duration		Type of test	Reading			
