# Supplementary material for: Providing Diabetes Education through Phone Calls Assisted in the Better Control of Hyperglycemia and Improved the Knowledge of Patients on Diabetes Management
Source: Healthcare (Basel). 2023 Feb 10;11(4):528. doi: 10.3390/healthcare11040528 (PMC9957542; doi:10.3390/healthcare11040528)
Supplement: Supplementary file 1 [file healthcare-11-00528-s001.zip › Supplemental information 9 - Knowledge Assessment Questions.pdf]

Knowledge Assessment Questions

1. Food can be consumed in diabetics in____________
   Full meals at once_____ Little food in regular intervals______
2. Physical activity in diabetics include
   Regular household works__ Exclusive physical activity like walking, cycling, yoga)__
3. Medications should be taken as prescribed by the doctor
   Yes, Always____ Dosage can be altered as per patients convenience___
4. Consumption of alcohol _________________ sugar levels
   Increases___ Decreases_____ Don't impact ______
5. Smoking _________ complications of diabetes
   Increases___ Decreases_____ Don't impact ______
6. Uncontrolled diabetes could lead to
   Heart diseases___ Eye problems___ Kidney problems ____ Stroke ____
   Skin diseases____ Foot ulcers____
7. Good glucose control value in random glucose testing should be <u>80-120 mg/dl</u>
   Answered_____ Not answered______
8. Investigation which is done once in every 3-4 months that gives average glucose range is called as <u>HbA1C</u>
   Answered_____ Not answered______
9. Good glucose control range is <u>6-7%</u> in HbA1C investigation
   Answered_____ Not answered______
10. Diabetes can be ________________ to keep glucose under control

    Self-managed____ managed through medications____ Can't manage anyways____

    * Each question carries 1 point.
    **For Question 6, give point even if only one answer is given. Mark the answer so that we have to give future education on rest of the answers
