# Supplementary figures and images for: Providing Diabetes Education through Phone Calls Assisted in the Better Control of Hyperglycemia and Improved the Knowledge of Patients on Diabetes Management
Source: Healthcare (Basel). 2023 Feb 10;11(4):528. doi: 10.3390/healthcare11040528 (PMC9957542; doi:10.3390/healthcare11040528)

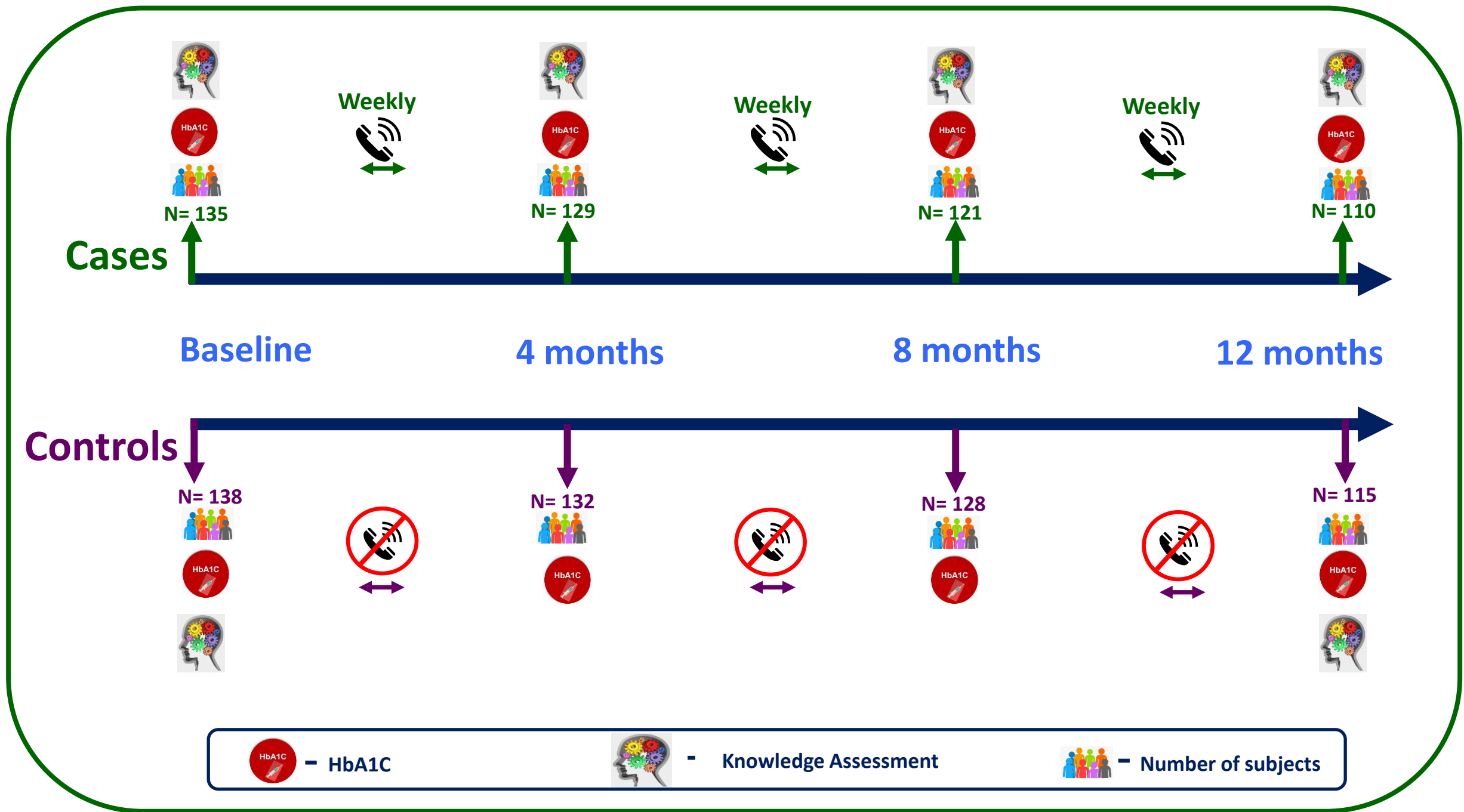

Supplement: Supplementary file 1 [file healthcare-11-00528-s001.zip › Supplemental information 4 - Schematic diagram of the stdy.pdf]
